# FCIQMC-CASPT2 with Imaginary-Time-Averaged Wave Functions

**DOI:** 10.1021/acs.jctc.4c01462

**Published:** 2025-01-17

**Authors:** Arta A. Safari, Robert J. Anderson, Ali Alavi, Giovanni Li Manni

**Affiliations:** † Max-Planck-Institute for Solid State Research, Heisenbergstraße 1, 70569 Stuttgart, Germany; ‡ Yusuf Hamied Department of Chemistry, University of Cambridge, Lensfield Rd, Cambridge CB2 1EW, United Kingdom

## Abstract

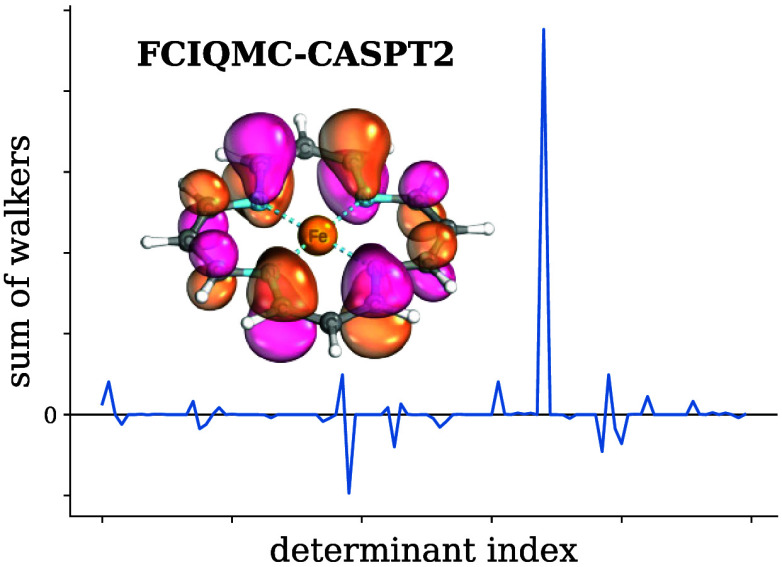

A new method to perform complete active space second-order perturbation theory on top of large active spaces optimized with full configuration quantum Monte Carlo is presented. Computing the three- and Fock-contracted four-particle density matrix from imaginary-time-averaged wave functions is found to resolve fermionic positivity violations and to ensure numerical stability. The protocol is applied to [NiFe]-hydrogenase, [Cu_2_O_2_]-oxidase and Fe-porphyrin model systems up to 26 electrons in 27 orbitals and benchmarked against DMRG-CASPT2.

## 1 Introduction

The last two decades have witnessed significant advances in solving the full configuration interaction (FCI) problem for large Hilbert spaces with sparse solvers like the density matrix renormalization group (DMRG),^1^ FCI Quantum Monte Carlo (FCIQMC),^2^ or other selected CI approaches.^3−5^ Despite their enabling power, all these methods are inefficient at converging dynamic correlation effects that require large orbital bases.^6^ Research has therefore focused on using large complete active space (CAS) wave functions capturing near-degeneracy effects as reference states for correlation methods that include the external space orbitals. Perturbative techniques are well suited to this end, because apart from an operator partitioning, they contain few if any empirical parameters and can treat large orbital expansions at reasonable cost.

One major issue with multiconfigurational perturbation theory is that a straightforward generalization of Møller–Plesset theory yields amplitude equations proportional to the length of the reference expansion. Reasonable CAS spaces easily exceed hundreds of millions of determinants, rendering the exact solution of the uncontracted approach infeasible for all practical purposes. One way to circumvent this limitation is internal contraction, i.e. variational degrees of the zeroth-order wave function are frozen under the perturbation, making the perturber space mostly independent of reference size.^7^ Dynamic correlation effects outside the active space are in this manner expressed as higher-order interactions within the CAS. The exponential complexity resurfaces in the associated active space reduced density matrices (RDMs), typically up to rank three and four, which are six and eight index tensors with computational cost  and storage requirements . In light of the active orbital dependence, even with a polynomial ansatz for the CI expansion (*n*_CAS_), these calculations can quickly exceed the capacities of contemporary computing architectures.

An alternative way forward is based on the realization that the perturber space, just like the CAS, is sparse and therefore amenable to stochastic sampling^8−10^ or tensor decompositions.^11,12^ Since the external space is considered explicitly, no higher-order RDMs are required, making them more easily compatible with large CAS references. Certain components of the perturbation equations are easier to treat in one framework than the other and hybrid contracted/uncontracted approaches have already been reported.^13,14^

Recently, the combination of FCIQMC with complete active space second-order perturbation theory (CASPT2)^15−18^ was reconsidered,^19^ due to its favorable cost-to-performance ratio compared to other operator partitionings. Already in the first study on stochastic internally contracted perturbation theory,^20^ the nonorthogonality of perturbers in CASPT2 caused high sensitivity to “noise” in the replica sampling^21,22^ of rank three and four RDMs. Contributions to the *n*-RDM are made by determinantal connections up to rank *n*; however, sampling triple and quadruple excitations nonuniformly is difficult, since they are not connected through the Hamiltonian. At the same time, higher-order excitations are more numerous than singles and doubles, leading to numerical instabilities in FCIQMC-CASPT2, if they are sampled uniformly.^20^ An improved, nonuniform sampling algorithm was hence deemed critical for a practical implementation.

Stochastic excitation generation is usually enhanced by deterministic projection in a subspace of highly weighted determinants, reducing the number of walkers required for a target error significantly. Within this semistochastic space (23) all determinantal connections of the Hamiltonian are accounted for exactly, which in a certain sense constitutes an optimal excitation generator. The question remained whether the semistochastic space could be repurposed to deterministically account for triple and quadruple contributions between highly populated determinants. Complementary to deterministic enumeration, higher-order contributions due to single and double connections, e.g. ⟨*D*_I_|*p*^+^*q*|*D*_J_⟩ → ΔΓ_*pq*,*rr*,*ss*_, were sampled nonuniformly in the remainder space. Even with these modifications, numerical instabilities in FCIQMC-CASPT2 persisted, the magnitude of which could be linked to fermionic positivity violations of the 3RDM across different initializations of the pseudorandom number generator (PRNG).^19^ While no systematic bias in FCIQMC was found that would explain the origin of the “noise”, accounting for sampling errors a posteriori by a convex relaxation to the closest positive-semidefinite 3RDM and averaging over multiple PRNG seeds proved sufficient to present a prototype in the pseudocanonical basis.^19^

**Figure 1:**
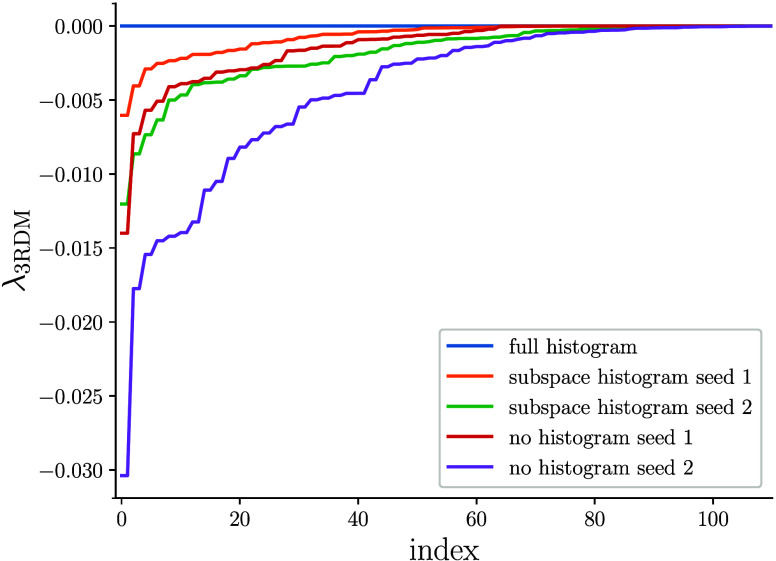
Negative eigenvalue distributions of 3RDMs obtained from FCIQMC calculations with 1 × 10^6^*N*_walker_ and a semistochastic space of 12.5k determinants after 20k iterations on the natural orbitals of the Cr_2_ valence CASSCF(12,12) at 1.9 Å distance. For both on-the-fly sampling schemes, two initializations of the PRNG are shown.

By definition, RDMs derived from a variationally optimized CI vector are fermionic. FCIQMC is a projective method where each instantaneous realization of the CI vector carries no physical meaning, but variational CI coefficients emerge as averages over imaginary time in the walker and sampling limit. Since determinants in the semistochastic space have to be kept in memory even if they are unoccupied, averaging the subspace coefficients (histogramming) comes at no appreciable overhead
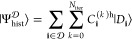
1where *C*_**i**_^(*k*)^ is the instantaneous walker weight on determinant |*D*_**i**_⟩ at iteration *k*. To combine this procedure with standard replica sampling,^21^ contributions from the deterministic subspace have to be normalized by the number of cycles that the determinant is occupied. In Figure 1, we compare the 3RDM eigenvalue distributions obtained with such hybrid semistochastic histogramming+replica sampling to the subspace approach from ref (19) without any histogramming. While partial histogramming lessens the negative eigenvalue tails, it does not cure the strong dependence on the PRNG seed and more importantly shows that the issue of RDM positivity violations cannot exclusively be traced back to nonuniform/exact excitation generation or a lack thereof. It also implies that negative eigenvalues stem both from within and outside the deterministic space, posing the question of whether a pure histogramming scheme without replica sampling is practical. Such a protocol incurs a larger truncation error than hybrid approaches, but as evidenced by Figure 1, leads to positive semi-definite RDMs.

The purpose of this paper is to introduce the histogrammed approach to RDMs as a new RDM algorithm and demonstrate that for active spaces up to 27 orbitals, it can be used to perform numerically well-conditioned FCIQMC-CASPT2. In section 2 we briefly outline the conventional CASPT2 method before considering the ingredients for FCIQMC-CASPT2 in section 3. Finally, three model systems relevant to transition metal chemistry are treated in the new framework in section 4: the singlet/triplet splitting in [NiFe]^2–^ hydrogenase, the isomerisation potential of [Cu(NH_3_)]_2_O_2_^2+^, and the triplet/quintet gap of a simplified Fe-porphyrin. DMRG-CASPT2 data is available for the first two compounds and serves as a reference.

## 2 CASPT2

Several didactic introductions covering the CASPT2 method already exist.^24,25^ Here we focus on the aspects most relevant for combining it with FCIQMC.

CASPT2 uses the generalized Fock operator projected onto the zeroth-order state as the unperturbed operator
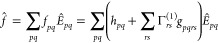
2here *h*_*pq*_ and *g*_*pqrs*_ are the one-/two-electron integrals respectively,  is the standard spin-free singlet excitation operator, and Γ_*rs*_^(1)^ is the 1RDM. Grouped in classes by the number of electrons in the inactive, active, and virtual orbitals, the first-order interacting space is spanned by nonorthogonal, overcomplete perturbers that are orthogonalized by diagonalization and inversion of their overlap matrix to ensure numerical stability. The metric tensors can be obtained from at most the normal-ordered 3RDM. Assuming real orbitals the latter is defined as
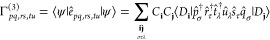
3where small Greek letters denote spin, and small Latin letters denote spatial orbital indices. Solving for the second-order energy also requires the contraction of the Fock matrix with the 4RDM (F.4RDM)
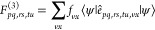
4

In the exact diagonalization regime, the invariance of the CASSCF wave function under active−active rotations is exploited to rotate |ψ⟩ into pseudocanonical orbitals, which diagonalizes eq 2 and removes one index from the summation in eq 4. Moreover, as the CAS is closed under excitations, it is advantageous to factor out the single excitation operator
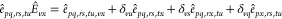
5and compute the F.4RDM as a transition 3RDM with the excited wave function |*F*⟩
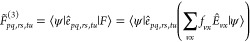
6Γ^(3)^ and  are sufficient to construct all remaining intermediates occurring in CASPT2. We have adapted the OpenMolcas/FCIQMC interface^19,26,27^ to be compatible with arbitrary orbital bases and use it to solve for the second-order energy.

## 3 FCIQMC and Reduced Density Matrices

FCIQMC belongs to the family of projector Monte Carlo techniques that integrate the imaginary time Schrödinger equation in Fock space. To obtain the extremal eigenvector of the Hamiltonian, a linearized projector is repeatedly applied onto a state that has nonzero overlap with the final solution. In FCIQMC, this matrix-vector product is reformulated in terms of a stochastic walker dynamic. The expectation value of the stochastic matrix multiplication coincides with the exact result, but unlike deterministic projection, only sparse, instantaneous snapshots of walkers are stored, resulting in lower memory requirements.

As the wave function in FCIQMC is not static, RDMs needed for gradients and properties are sampled on-the-fly by calculating the expectation value
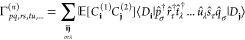
7

Since the expectation value of a product is not equal to the product of expectation values unless the quantities are uncorrelated, particularly the instantaneous diagonal entries give rise to a bias that also affects off-diagonal elements through trace normalization
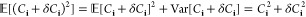
8

Introducing an independent replica,^21,22^ denoted by the numbers in round brackets, guarantees zero covariance between the coefficients and provides access to the exact RDM in the infinite walker and sampling limit. The appeal of this approach is that a polynomial number of Γ_*pq*,*rs*,*tu*,···_^(*n*)^ elements are averaged rather than an exponentially growing set of CI coefficients.

Whereas diagonal contributions correspond to sums of products of instantaneous occupations, off-diagonal terms are sampled in the spawning step of FCIQMC, which already generates connections **i** → **j**. Nonzero RDM contributions for such a spawning event in replica one require *C*_**j**_^(2)^ ≠ 0 and vice versa, because *C*_**i**_^(1)^*C*_**j**_^(2)^ otherwise vanishes. This way, it can be seen how the overlap between replica one and two affects sampling efficiency.

RDMs obtained with the replica approach are quasivariational in the sense that on average both replicas sample the same wave function. Over shorter imaginary time evolutions, stochastic errors cause ⟨ψ^(1)^|ψ^(2)^⟩ to be smaller than one, meaning that the RDM positivity conditions cannot be fulfilled exactly. This may also explain why fermionic positivity violations were found to correlate strongly with the hermiticity error and become more severe with higher RDM rank:^19^ as the number of appreciable contributions rises, the replica overlap has to increase to accrue nonzero elements. In practice, the positivity violations cause no problems if the theory is linear in the RDM, e.g. CASSCF or strongly contracted NEVPT2.^20^ However, for methods like CASPT2, containing nonlinear steps such as Löwdin orthogonalization of the perturber overlap, strict positivity is important to ensure numerical stability. This requirement provides added impetus to devise a scheme that guarantees strict positivity by construction.

Another way to obtain an unbiased *C*_**i**_*C*_**j**_ product is to first average the respective coefficients to obtain an uncorrelated estimate before multiplying them. While averaging the occupation of every visited determinant in a dynamic is infeasible, it was previously not considered whether a representative subset of important states could be identified, histogrammed, and used to calculate RDMs. A consequence of considering a subset is that in the walker and sampling limit, the obtained wave function is a renormalized projection of the exact CI vector onto the histogrammed determinants. Projection in this sense is not equivalent to solving the projected Schrödinger equation, as for instance in Coupled-Cluster theory. In this context, it had been observed that RDMs computed by means of response theory can violate fermionic positivity.^28,29^ Even though they do not derive from subspace diagonalization as in selected CI,^5,30^ RDMs from histogrammed wave functions are nevertheless variational, as they are eigenfunctions of the effective Hamiltonian
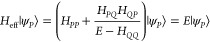
9where *P* and *Q* denote the histogrammed and remaining sets, respectively. Computing the effective integrals for *H*_eff_ from *H*_*PP*_ is not straightforward, and for accurate results, the coupling between the histogrammed and external space must be weak, i.e. the overlap of the exact with the histogrammed wave function should be >99%. Consequently, the histogrammable set has to capture a large fraction of the wave function, as measured by the *L*_1_ norm at every iteration. Selections from instantaneous estimates, like the final population of a converged calculation, do not contain any dynamic information and are poorly suited to this end. Instead we propose a greedy algorithm, where after reaching stationary dynamics, a determinant occupied for one reporting period with a certain number of walkers is added to the histogrammable set. This selection scheme is conceptually simple but has two flaws: (i) in the sampling limit, every determinant with nonzero ground state overlap fulfills the occupation criterion and (ii) many unimportant determinants will be included. As demonstrated in the next section, sampling for roughly one thousand iterations from an already converged population was sufficient to make meaningful selections in the cases considered here. Determinants with average occupation lower than a specific threshold, denoted *t* in units of walkers *N*_walker_, can be discarded in the subsequent RDM formation, rendering the overhead of including superfluous determinants at the histogramming step negligible in practice.

**Figure 2:**
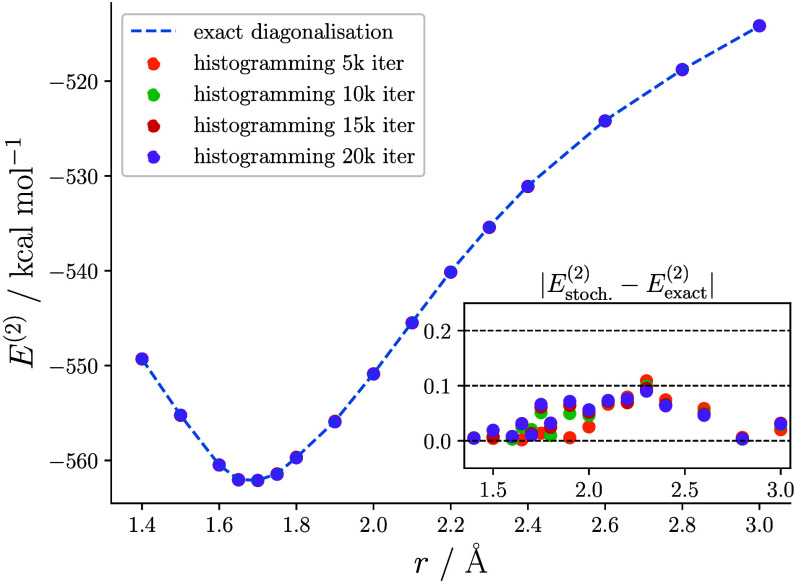
Chromium dimer CASSCF(12,12)/CASPT2 binding curve with an imaginary shift of 0.2 *E*_h_ and IPEA of 0.45 *E*_h_ obtained by histogramming a 12.5k determinant semistochastic space for varying durations. CASSCF(12,12) natural orbitals, 10^6^*N*_walker_ and the *C*_1_ point group were employed to mimic the parameters of ref (19).

The averaging period needed for an accurate histogram depends on the walker number and autocorrelation time. As shown in Figure 2, numerical tests on the chromium dimer CAS(12,12) suggest that a few thousand iterations are sufficient to produce numerically stable CASPT2 results for small systems.

For larger active spaces and smaller time steps, longer durations can be expected. Unless noted otherwise, the results presented in the next section are obtained with averaging durations between 10k and 15k iterations. Details are provided in the Supporting Information.

**Figure 3:**
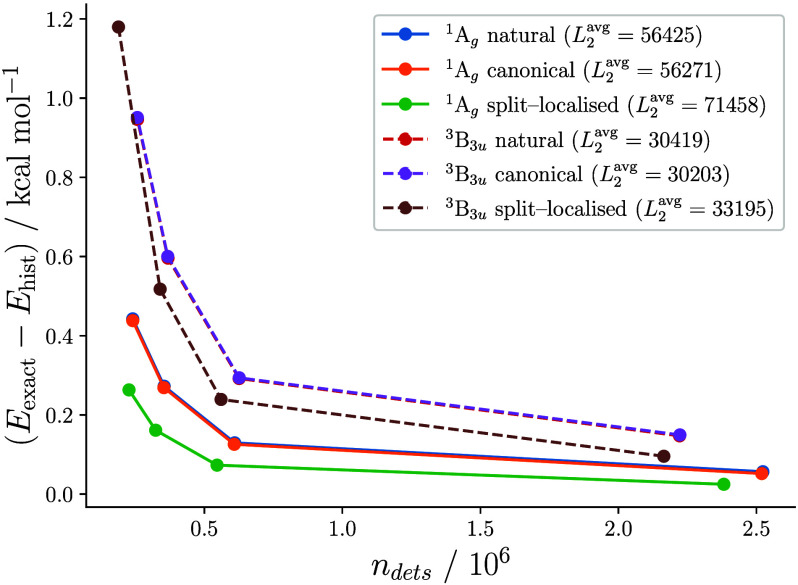
Convergence of the absolute histogrammed CASCI energy in different orbital bases for the lowest ^1^A_*g*_ and ^3^B_3*u*_ states of anthracene. The entire π system is correlated in the CAS(14,14). Optimized singlet and triplet geometries were taken from ref (36). Compared to the singlet with a reference weight of 73%, the triplet is more strongly correlated, having two dominant configurations with 31% and 27% weight, respectively. FCIQMC calculations used 5 × 10^6^*N*_walker_ and *D*_2*h*_ symmetry. Average *L*_2_ norms were computed by blocking analysis.^37^

That the convergence of the wave function under truncation varies depending on the single-particle basis has been known for decades^31^ and is exploited ubiquitously in sparse solvers.^5,30,32,33^ Split-localization of the natural orbitals yields correlated orbital pairs located in the same part of the molecule. The lowest correlating orbital is similar in shape to its bonding counterpart with an additional nodal plane at the center.^34^ It has been shown that for the same number of determinants, these orbitals typically display superior energy convergence over natural orbitals in the chemically relevant interval up to ∼0.1 kcal mol^–1^.^35^ In Figure 3, we illustrate the effect of the single-particle basis on absolute energy convergence in the histogramming scheme, correlating the π system of anthracene in a CAS(14,14).

As can be seen from the 20–30% higher averaged *L*_2_ norms, split-localization compactifies the space of sparsely populated determinants and enables a reduction of the histogrammed space up to a factor of five compared to natural orbitals while providing equivalent or better variational energies. Even though in almost all cases presented here also a lower variance of the projected energy estimator was observed, it is important to note that split-localized orbitals do not guarantee the sparsest CI solution in the *L*_1_ sense, e.g. exact diagonalization of the ^3^B_3*u*_ state yields ∥ψ∥_1_^nat.^ = 84.4, ∥ψ∥_1_^can.^ = 84.6, and ∥ψ∥_1_^split^ = 87.7. The negligible difference between canonical and natural orbitals is probably due to the high point group of anthracene.^38^ We found the benefit of split-localized orbitals to increase with active space and molecular size and therefore used them in all presented calculations. Whether it is beneficial to sacrifice symmetry in the localization procedure has to be decided on a case by case basis. In our calculations, the highest abelian point group of the molecule was preserved.

Computing RDMs from large histogrammed sets efficiently requires dedicated algorithms, due to a lack of structure in stochastic selections. Here, we extend the algorithm originally devised in the context of semistochastic heat-bath CI^39^ to the 3RDM and F.4RDM. Details on our implementation and strategies to compress the excited wave function |*F*⟩ are given in the Appendix.

## 4 Application

In this section, three examples from transition metal chemistry in order of increasing active space size are presented to assess the new formulation of FCIQMC-CASPT2: the singlet/triplet gap of a [NiFe]^2–^ hydrogenase model, the isomerization potential of [Cu(NH_3_)]_2_O_2_^2+^, and the triplet/quintet gap of an Fe-porphyrin model. DMRG-CASPT2 data without cumulant-approximated F.4RDM^38^ is available for the [NiFe]^2–^ and [Cu(NH_3_)]_2_O_2_^2+^ compounds from a previous study^40^ and serves as a reference to gauge the expected accuracy of our protocol. Across all calculations we use IPEA^17^ and imaginary shifts^18^ of 0.25 *E*_h_ and 0.1 *E*_h_, respectively.

### 4.1 Singlet/Triplet Gap of a [NiFe]^2–^ Hydrogenase Model

Hydrogen gas is a prospective candidate for carbon-free fuels. Because they catalyze the pertinent conversion between hydrogen gas and protons and electrons, hydrogenases have been studied with both experiments^41−45^ and calculations.^40,46−49^ Here we consider the singlet/triplet gap of the dinuclear nickel iron hydrogenase model [NiFe]^2–^ with *C*_*s*_ symmetry. The singlet and triplet states belong to ^1^*A*′ and ^3^*A*″^40,47^ and are described with a CAS(20,21) and CAS(22,22), respectively, containing the Ni 3*d*, 3*d*′, Fe 3*d*, three of the 3*d*′ and three (^1^*A*′) or four (^3^*A*″) bonding ligand orbitals.^47^ With the ANO-RCC basis contracted to Fe/Ni(21s15p10d6f4g2h)/[7s6p5d3f2g1h], S(17s12p5d4f)/[5s4p2d1f], C/N/O(14s9p4d3f)/[4s3p2d1f], and H(8s4p)/[3s1p], the reported estimates for Δ*E* = *E*(^3^*A*″) – *E*(^1^*A*′) are −15.8 kcal mol^–1^ at DMRG-CASSCF and 14.7 kcal mol^–1^ at DMRG-CASPT2 level of theory.^40^ The DMRG-CASSCF gap can be reproduced with the replica 2RDM approach using 50 × 10^6^*N*_walker_, and we use the same number for the histogram. As shown in Table 1, convergence to the replica CASSCF gap is rapid, even with a small number of retained determinants. For the chosen histogrammable space, increasing the number of included determinants leads to a FCIQMC-CASPT2 estimate of 15.7 kcal mol^–1^, which is within chemical accuracy of the best DMRG-CASPT2 estimate and just 0.3 kcal mol^–1^ from cumulant-approximated DMRG-CASPT2. As shown in section S2 of the Supporting Information for the Chromium dimer CAS(12,12), there is reason to believe that these deviations stem primarily from slower convergence of the 3RDM with increasing histogram space size. The appeal of histogramming is that this error becomes systematically controllable and does not void numerical stability of FCIQMC-CASPT2, even under noisy conditions. Overall, the remaining error is a fraction of the very large differential correction (>30 kcal mol^–1^), and the correct singlet ground state is obtained.

**Table 1:** Singlet/Triplet Splitting Δ*E* = *E*(^1^*A*′) – *E*(^3^*A*″)/kcal mol^–1^ at FCIQMC-CASSCF/CASPT2 Level of Theory with 50 × 10^6^*N*_walker_ for Different Discard Thresholds *t*/*N*_walker_^a^

*t*	FCIQMC-CASSCF	FCIQMC-CASPT2	*n*_det_(^1^*A*′)	*n*_det_(^3^*A*″)
3.0	–15.9	15.5	1.9	1.8
2.0	–15.9	15.6	3.0	2.8
1.0	–15.8	15.6	6.5	6.3
0.5	–15.8	15.7	13.0	13.0

a Number of retained determinants in the truncated wave functions in units of millions.

### 4.2 Isomerization Gap of Copper Oxide [Cu(NH_3_)]_2_O_2_^2+^

Copper enzymes are common in nature, with examples ranging from tyrosinase to catechol oxidase or hemocyanin.^50−52^ At their active site, these enzymes feature a  core in a variety of isomers. Here we consider the isomerization barrier between the singlet bis(μ-oxo) and μ-η^2^: η^2^-peroxo forms, which is known to be a challenging problem for quantum chemistry.^153−55^ This particular pair has been considered in a number of previous theoretical studies.^40,53−56^ Quantitative modeling was suggested to require a 28 orbital active space comprising the Cu 3*d*, 3*d*′, the in-plane O 2*p*, 2*p*′, as well as a proper treatment of relativistic and solvent effects. We restrict ourselves to the CAS(24,24), neglecting the O 2*p*′ double shell for both structures to be able to compare our results to DMRG-CASPT2 data.^40^ Choosing the ANO-RCC basis with contractions Cu(21s15p10d6f4g2h)/[7s6p5d3f2g1h], N/O(14s9p4d3f)/[4s3p2d1f], H(8s4p)/[3s1p] and this active space, the best estimates for Δ*E* = *E*(bis(μ-oxo)) – *E*(peroxo) are 19.5 kcal mol^–1^ at DMRG-CASSCF and 22.6 kcal mol^–1^ at DMRG-CASPT2 level of theory.^40^ Replica 2RDM energies are converged with 100 × 10^6^*N*_walker_, and we use the same number for histogramming.

Convergence to the reference CASSCF gap within 0.1 kcal mol^–1^ accuracy is still fast, as shown in Table 2, yet slower than that for [NiFe]^2–^, as the wave functions are more diffuse. FCIQMC-CASPT2 energies converge to 21.9 kcal mol^–1^, which is in good agreement with exact DMRG-CASPT2, but closer to 22.2 kcal mol^–1^ reported in ref (40) with cumulant-approximated F.4RDM.

**Table 2:** Isomerization Potential Δ*E* = *E*(bis(μ-oxo)) – *E*(peroxo)/kcal mol^–1^ at FCIQMC-CASSCF/CASPT2 Level of Theory with 100 × 10^6^*N*_walker_ for Different Discard Thresholds *t*/*N*_walker_^a^

*t*	FCIQMC-CASSCF	FCIQMC-CASPT2	*n*_det_(peroxo)	*n*_det_(bis(μ-oxo))
3.0	19.8	22.1	3.9	3.7
2.0	19.7	22.0	6.2	5.7
1.0	19.6	21.9	14.0	13.0
0.5	19.6	21.9	27.0	27.0

a Number of retained determinants in the truncated wave functions in units of millions.

### 4.3 Triplet/Quintet Gap of Iron Porphyrin Model Fe(P)

Derivatives of porphyrin occur in many biological systems and take key roles in photosynthesis or oxygen transport.^57^ A better understanding of their electronic structure promises to yield deeper insights into these important processes.

The previous benchmarks on [NiFe]^2–^ and [Cu(NH_3_)]_2_O_2_^2+^ suggest that the accuracy of FCIQMC-CASPT2 with histogramming is comparable to DMRG-CASPT2 with cumulant-approximated F.4RDM. In this section, we consider an active space for which no reference data is available. We employ the same structure already used in previous studies^59,63^ and contract the ANO-RCC basis set to Fe(21s15p10d6f4g)/[6s5p3d2f1g], C/N(14s9p4p3f)/[4s3p2d1f], and H(8s4p3d)/[3s2p1d]. Full valence CASSCF(32,34) calculations had established that the ^3^*E*_*g*_ is in vacuum more stable than the ^5^*A*_*g*_ by 3.5 kcal mol^–1^.^59^ Explicitly accounting for Fe 3*s*3*p* semicore correlation, a known weakness of CASPT2,^58,60^ in a CAS(40,38) further increases the gap to 4.4 kcal mol^–1^.^63^ An uncontracted MRCISD-like treatment on top the CAS(32,34) reference in a truncated orbital space of 32 inactive and 93 virtual orbitals via the generalized active space framework yields a spin gap of 7.0 kcal mol^–1^.^61^ While uncontracted MRCI is usually very accurate, a drawback is the limited number of orbitals that can be correlated. Particularly the virtual orbitals exhibit no clear plateau, and it remains unclear how many of them have to be included to obtain converged results. Internally contracted perturbation theory is less limited in the number of correlated orbitals, and we use our implementation to assess whether a second-order correction on a large reference is sufficient to obtain the correct ordering of states.

Due to the rapid growth of the excited wave function, converging the F.4RDM of a CAS(32,34) reference is not feasible and calls for a reduction of the active space. Based on the natural occupation numbers of the CAS(32,34), see Figures 3 and S2 of ref (59), and the active space construction logic of the previous sections, we include five Fe 3*d*, five radially correlating 3*d*′, the 16 orbitals of the π system, and one bonding σ_Fe-N_ into the active space, yielding a CAS(26,27). Natural occupation numbers of the excluded Fe 3*s*′, 3*p*′ and three bonding σ_Fe-N_ orbitals with additional nodes deviate by at most 0.03 electrons from zero suggesting that their influence can be described perturbatively. After orbital optimization, the quintet is stabilized over the triplet by Δ*E* = *E*(^5^*A*_*g*_) – *E*(^3^*E*_*g*_) = −8.8 kcal mol^–1^. In the split-localized basis, this gap can be reproduced with 125 × 10^6^*N*_walker_ using the replica approach, see Table 3, which we accordingly used for histogramming.

**Table 3:** Quintet/Triplet Gap Δ*E* = *E*(^5^*A*_*g*_) – *E*(^3^*E*_*g*_)/kcal mol^–1^ at FCIQMC-CASSCF/CASPT2 Level of Theory with 125 × 10^6^*N*_walker_ for Different Discard Thresholds *t*/*N*_walker_^a^ Compared to RASSCF/CI+RASPT2^b^

method	*t*	MCSCF	MCPT2	*n*_det_(^5^*A*_*g*_)	*n*_det_(^3^*E*_*g*_)
replica		–8.8			
histogram 15k iterations					
	3.0	–9.7	–2.5	5.5	5.8
	2.0	–9.4	–2.4	8.6	9.4
	1.0	–9.2	–2.3	18.0	21.0
	0.5	–9.0	–2.2	36.0	42.0
	0.25	–9.0	–2.2	61.0	68.0
histogram 30k iterations					
	3.0	–9.6	–2.5	5.5	5.9
	2.0	–9.4	–2.4	8.6	9.5
	1.0	–9.1	–2.3	18.0	21.0
	0.5	–9.0	–2.2	36.0	41.0
	0.25	–9.0	–2.2	60.0	67.0
RASSCF(2h, 2p)		–12.0	–6.5	0.3	0.8
RASSCF(3h, 3p)		–9.8	–1.9	16.0	32.0
RASCI(3h, 3p)		–15.0	–6.5	16.0	32.0
RASCI(3h, 4p)		–6.7	–2.7	135.0	257.0

a Number of retained determinants in the truncated wave functions in units of millions.

b To improve legibility, the abbreviation RAS(x, y) = RAS(26, x, y; 9, 6, 12) is used; see the main text for details.

At *t* = 0.5 *N*_walker_, the variational energy is converged to −9.0 kcal mol^–1^, which is within 0.2 kcal mol^–1^ of the replica CASSCF result. FCIQMC-CASPT2 decreases this gap to −2.2 kcal mol^–1^, but it is insufficient to invert the ordering. Because further reduction of the discard threshold to *t* = 0.25 did not influence either the CASCI or PT2 gaps, the role of histogramming duration was scrutinized by doubling it from 15k to 30k iterations; however, the gaps remain unchanged.

For the lack of a better reference, RASPT2 calculations^62^ were performed with nine π/Goutermann orbitals in RAS1, five Fe 3*d* and one bonding σ_Fe-N_ in RAS2 and the remaining 12 orbitals in RAS3, denoted RAS(26, x, y; 9, 6, 12) or RAS(x, y) for short. RASCI(3h, 3p)+PT2 calculations on the FCIQMC-CASSCF orbitals exhibit poor convergence in terms of absolute and relative energies, being 5.8 kcal mol^–1^ and 4.3 kcal mol^–1^ above the best estimates for the spin gap, respectively. When the excitation level is increased to RASCI(3h, 4p)+PT2, the limit of what is feasible with exact diagonalization, absolute energies are still higher than the histogrammed ones by more than 32 m*E*_h_, but due to error cancellation, lower RASCI and RASPT2 gaps of −6.7 kcal mol^–1^ and −2.7 kcal mol^–1^ are obtained. Reoptimizing the orbitals with RASSCF yields more reasonable spin gaps with −9.8 kcal mol^–1^ at RASSCF and −1.9 kcal mol^–1^ at RASPT2 level of theory; however, the quintet remains in the ground state.

In light of the qualitative agreement between exact diagonalization and FCIQMC, we conclude that a decisive stabilization of the triplet over the quintet, analogous to the [NiFe]^2–^ model, cannot be expected with CASPT2 on this active space. Larger active spaces like the CAS(32,34)^59^ thus remain necessary to obtain a suitable reference wave function for perturbation theory and the correct spin state energetics. Relaxation effects under dynamic correlation are also included at fourth order of perturbation,^64,65^ but in an internally contracted framework even higher rank RDMs would be required.

## 5 Conclusion

We have presented an improved version of FCIQMC-CASPT2 based on a novel procedure to calculate higher-order density matrices from imaginary-time-averaged wave functions. The new strategy ameliorates fermionic positivity violations arising in the replica RDM approach and enables numerically stable FCIQMC-CASPT2 calculations. Examples from transition metal chemistry up to 26 electrons in 27 orbitals suggest that the fidelity of the protocol is within chemical accuracy of full DMRG-CASPT2, yet closer to the formulation with cumulant-approximated F.4RDM.

Overall, we consider the histogramming approach not as a replacement for replica sampling, but rather complementary whenever nonlinear operations on RDMs need to be performed. Future investigations should consider the optimization of perturber classes that give rise to higher-order RDMs in an uncontracted framework,^8^ which is more amenable to stochastization. Moreover, although the present study does not convey optimism that the RDM positivity conditions can be fulfilled exactly through improved excitation generation, it may be possible to modify the replica sampling in a manner that lessens the symptoms to a palatable degree.

## Figures and Tables

**Figure 4:**
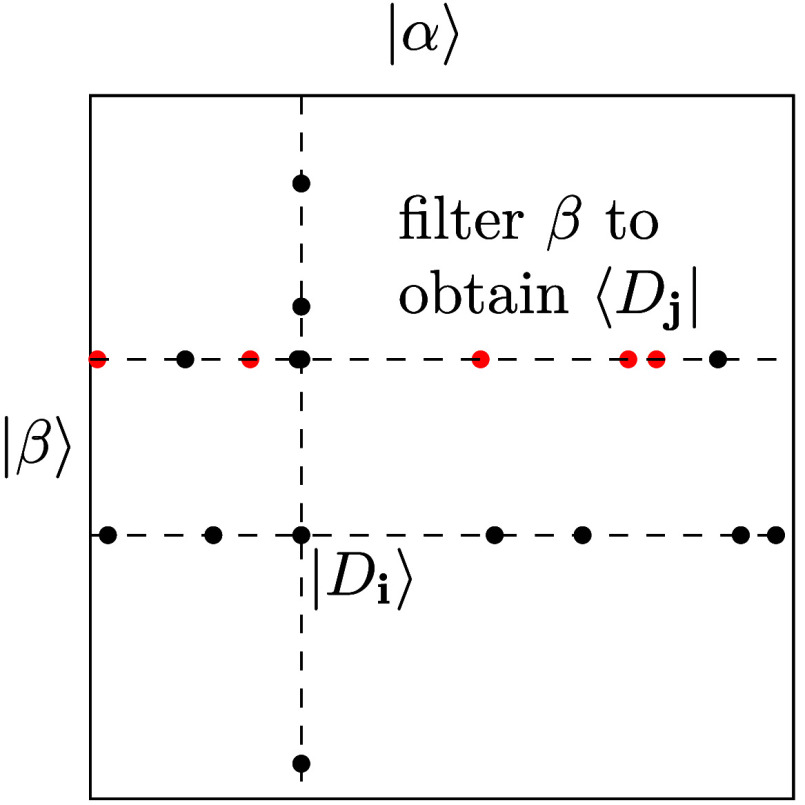
Visual aid to the spin map algorithm. The unstructured set is first converted into a list of unique α and β strings. Arranged in matrix form, such that every point in the rectangle represents a determinant, many strings will be missing, since the histogrammed wave function is a subset of the FCI. See text for details.

## Appendix: CASPT2 Intermediates from Sparse Sets

According to eq. 3, spin-free RDMs can be decomposed into spin-resolved components, e.g.

10

From this form, it becomes evident that two classes of excitations must be considered for a RDM of rank two or higher: terms of pure spin, like *αααα*, and mixed spin, such as *ααββ*. If |ψ⟩ is closed under excitations and represented in Slater determinants, the minimal operation count (MOC) method^66^ gives an asymptotically optimal algorithm to find all contributing pairs. Since α excitation operators, , leave β strings, *I*_β_, invariant and vice versa, factorization of the integrand is possible

11

and contributing determinants can be addressed with lexical indexing from a given source after the action of single and double excitation operators.^66^ Counting the number of possible excitations, the cost of computing the 2RDM is equivalent to a Direct-CI iteration for a two-body operator

12

where *n*_det_ is the number of determinants, *n*_orb_ is the number of spatial orbitals, and *n*_α/β_ is the number of α or β electrons. Unlike complete or restricted active space wave functions, the stochastic selection of the histogrammed set removes the closure property under excitations and prevents application of the MOC algorithm. This problem is already known from selected CI and can be solved by storing the connectivity within the sparse set. In the case of semistochastic heat-bath CI, connections are encoded in a number of precomputed and sorted arrays that allow to approach the MOC lower bound for the rate-determining mixed-spin excitations.^39^ Before turning to implementation details, it is instructive to visualize the general idea for the 2RDM as shown in Figure 4.

Every determinant is a unique combination of α and β strings and corresponds to a point in the rectangle spanned by these axes. For a given index combination, an efficient RDM algorithm has to find all contributing pairs (red dots) from a source determinant while visiting the lowest possible number of vanishing terms (black dots). Pure−spin excitations leave the complementary string invariant and can be resolved by checking all points in a single column or row for their excitation level with respect to the reference. Despite being asymptotically suboptimal, a single column/row is sufficiently sparse for this step to be fast. Moreover, rank *n* connectivity does not have to be stored to obtain contributions to the *n*-RDM. On the other hand, mixed-spin excitations involve multiple rows and columns that need to be traversed sparsely in order to approach MOC scaling. Rather than iterating over the entire column α, one can look up which of the existing α strings belongs to the set of single excitations from the reference. Each of these rows contains β strings that are not single β excitations from the reference, necessitating the use of a filter function. Whenever a valid contribution has been found, the combination of spin strings can be converted into a determinant address, and the respective CI coefficient product is added to the RDM.

In practice, four hash maps for α and β respectively are used in the implementation:

1. DetsContainAlpha: Given an α string retrieve the index of all determinants containing it in the histogrammed set. Retrieved indices are sorted in correspondence with the complementary string (beta for alpha) of the determinants encoded by the indices, i.e. to sort the indices, the β strings of the respective determinants are compared.
2. BetaWithAlpha: Given an α string retrieve all complementary β strings that occur with it in the histogrammed set.
3. AlphaSingleHoles: Map from (*N* – 1)-electron strings to all *N*-electron strings in the histogrammed set that can be generated from it.
4. AlphaSingleExc: From an α string, retrieve all strings which are connected by a single excitation. Given AlphaSingleHoles, it is already known which *N*-electron strings share a (*N* – 1)-string; consequently, this map can be quickly generated by enumerating all pairs of *N*-electron strings in a given bucket.

The filter step for mixed-spin excitations can be realized in linear time by intersecting the presorted BetaWithAlpha and BetaSingleExc buckets. Because elements in DetsContainBeta are sorted corresponding to BetaWithAlpha, matching elements can quickly be translated into a determinant address.

Generalizing the prescription above to the 3RDM requires storing all α and β doubles for mixed spin triples like Γ_*pq*,*rs*,*tu*_^*αα*,*αα*,*ββ*^. The associated memory cost is . Numerical experience suggests an increase in unique spin strings between  and . To speed up the construction of the hash tables, a similar (*N* – 2)-string list is constructed beforehand, but an additional filter step has to be included, as both singles and doubles share a (*N* – 2)-electron string. Based on scaling arguments of the MOC method for three body operators, a cost increase of one order of magnitude can be expected and is observed in practice

13

Taking inspiration from FCI codes,^27^ the F.4RDM is computed as a transition 3RDM, see eq. 6 in the main text, which avoids storage of triple connectivity within the histogrammed set. Since the set of determinants in the histogrammed wave function is not closed under single excitations, forming the excited wave function |*F*⟩ adds new determinants to the set that require the construction of new lookup tables. Note that this contraction runs over all excitations operators *Ê*_*vx*_, including diagonal number operators *Ê*_*vv*_, ensuring the excited wave function is a strict superset of the original wave function. This means that only a single set of lookup tables analogous to the ones for the 3RDM has to be stored, since the connectivity in the superset can be queried with a loop over all constituents of the subset. Depending on the number of spatial orbitals and point group symmetry, |*F*⟩ contains around one order of magnitude more elements than |ψ_hist_⟩, quickly rendering storage of the double connections prohibitive. Similar to what has been proposed for DMRG-CASPT2^38^ and DMRG-NEVPT2,^67^ the excited wave function is therefore compressed by removing determinants with a small *L*_1_ norm. Given that the excited wave function is typically one order of magnitude larger than the histogrammed set, the discard threshold is lowered by a factor of ten respectively. Using this approach, the discarded *L*_2_ norm of the excited wave function is usually less than one percent, and the errors in the PT2 energy are negligible, on the order of microhartrees. Additional experience with the method is needed to judge whether |*F*⟩ can be truncated more aggressively. If that were the case, it would significantly decrease the memory bottleneck of storing double connections in this set.

With the current implementation as described above, finding all contributing pairs in the most expensive 3RDM formation for the triplet state of Fe-porphyrin from a 67.6 million determinant histogrammed set took about 5 million core hours on two AMD EPIC 9554 (64 cores each) without simultaneous multithreading and can be completed with less than 512 GB of RAM. The same procedure for the F.4RDM took roughly 16.6 million core hours of two AMD EPYC 7763 CPUs (64 cores each) without simultaneous multithreading and requires approximately 1.5TB of RAM. Overall, these numbers give a rough estimation of the calculation cost, but should be taken with a grain of salt. As the goal of this study was to gauge the applicability of the histogramming approach, we made use of the C++ Standard Template Library and already existing self-written data structures whenever possible. Particularly the hash map implementation can be further optimized in terms of memory footprint and speed, which will be the subject of future work.

## Supporting Information Available

(A) absolute energies, (B) convergence analysis of RDMs in the histogramming scheme, (C) input files for calculations presented in the main section, and (D) orbitals in OpenMolcas format (ZIP)
