# Supplementary material for: FCIQMC-CASPT2 with Imaginary-Time-Averaged Wave Functions
Source: J Chem Theory Comput. 2025 Jan 17;21(3):1029–38. doi: 10.1021/acs.jctc.4c01462 (PMC11823415; doi:10.1021/acs.jctc.4c01462)
Supplement: Supplementary file 1 — ct4c01462_si_001.zip [file ct4c01462_si_001.zip › si/supplement.pdf]

Supporting Information: FCIQMC-CASPT2 with Imaginary-Time-Averaged Wave Functions

Arta A. Safari,*,† Robert J. Anderson,†,¶ Ali Alavi,†,‡ and Giovanni Li Manni*,†

†*Max-Planck-Institute for Solid State Research, Heisenbergstraße 1, 70569 Stuttgart, Germany*

‡*Yusuf Hamied Department of Chemistry, University of Cambridge, Lensfield Rd, Cambridge CB2 1EW, United Kingdom*

¶*Current address: Quantinuum, Terrington House, 13-15 Hills Rd, Cambridge CB2 1NL, United Kingdom*

E-mail: a.safari@fkf.mpg.de; g.limanni@fkf.mpg.de

Contents

S1 Absolute Energies **S-2**

S2 Convergence of RDMs in the Histogramming Scheme **S-4**

S3 Input files **S-5**

S3.1 Hydrogenase Model $[NiFe]^{2-}$ S-5

S3.1.1 Singlet S-5

S3.1.2 Triplet S-8

S3.2 Copper Oxide $[(CuNH_3)]_2\,O_2{}^{2+}$ S-12

S3.2.1 Bis(μ-oxo) S-12

S3.2.2 Peroxo S-15

S3.3 Fe-Porphyrin Model Fe(P) S-19

S3.3.1 Triplet S-19

S3.3.2 Quintet S-22

S1 Absolute Energies

Table S1: Absolute energies in E_h obtained from FCIQMC-CASSCF/CASPT2 with the histogramming approach for different discard thresholds t/N_walker on the $^1A'$ and $^3A''$ structures of $[\mathrm{NiFe}]^{2-}$.

t	$E_\mathrm{var}(^1A')$	$E_\mathrm{PT2}(^1A')$	$E_\mathrm{var}(^3A'')$	$E_\mathrm{PT2}(^3A'')$
3.0	-4685.29964000	-4688.52558906	-4685.32502500	-4688.50084494
2.0	-4685.29985500	-4688.52577086	-4685.32517300	-4688.50095001
1.0	-4685.30008100	-4688.52596455	-4685.32532700	-4688.50105357
0.5	-4685.30018100	-4688.52604946	-4685.32539500	-4688.50109242

Table S2: Absolute energies in E_h obtained from FCIQMC-CASSCF/CASPT2 with the histogramming approach for different discard thresholds t/N_walker on the bis(μ-oxo) and μ-η^2:η^2 peroxo isomers of $[(\mathrm{CuNH_3})_2\,\mathrm{O_2}]^{2+}$.

t	E_var(bis(μ-oxo))	E_PT2(bis(μ-oxo))	E_var(peroxo)	E_PT2(peroxo)
3.0	-3568.52290400	-3570.79456328	-3568.55450900	-3570.82975980
2.0	-3568.52323400	-3570.79485638	-3568.55469100	-3570.82993945
1.0	-3568.52358700	-3570.79517004	-3568.55488400	-3570.83014132
0.5	-3568.52373500	-3570.79530466	-3568.55497900	-3570.83024367

Table S3: Absolute energies in E_h obtained from FCIQMC-CASSCF/CASPT2 with the histogramming and replica sampling approaches for different discard thresholds t/N_walker, as well as RAS(26, x, y; 9, 6, 12) = RAS(x, y) type calculations on the ^5A_g and ^3E_g states of the iron porphyrin model complex.

method	t	$E_\mathrm{var}(^5A_g)$	$E_\mathrm{PT2}(^5A_g)$	$E_\mathrm{var}(^3E_g)$	$E_\mathrm{PT2}(^3E_g)$
replica					
		-1951.25034100		-1951.23636700	
histogram 15k					
	3.0	-1951.24902800	-1954.33251572	-1951.23364000	-1954.32845894
	2.0	-1951.24940000	-1954.33255656	-1951.23439100	-1954.32868410
	1.0	-1951.24980700	-1954.33257741	-1951.23521600	-1954.32890284
	0.5	-1951.25000800	-1954.33255925	-1951.23560600	-1954.32898364
	0.25	-1951.25008500	-1954.33254466	-1951.23574400	-1954.32901112
histogram 30k					
	3.0	-1951.24907000	-1954.33254470	-1951.23372100	-1954.32851932
	2.0	-1951.24944400	-1954.33259065	-1951.23447500	-1954.32875239
	1.0	-1951.24985600	-1954.33262023	-1951.23531200	-1954.32898727
	0.5	-1951.25006500	-1954.33261122	-1951.23571800	-1954.32908453
	0.25	-1951.25014800	-1954.33260196	-1951.23586600	-1954.32912065
exact diag.					
RASSCF(2h, 2h)		-1951.19035146	-1954.32348601	-1951.17196075	-1954.31315265
RASSCF(3h, 3p)		-1951.20739978	-1954.33532264	-1951.19176387	-1954.33229144
RASCI(3h, 3p)		-1951.19793269	-1954.33132714	-1951.17448446	-1954.32092904
RASCI(3h, 4p)		-1951.21415748	-1954.33267811	-1951.20341950	-1954.32837094

S2 Convergence of RDMs in the Histogramming Scheme

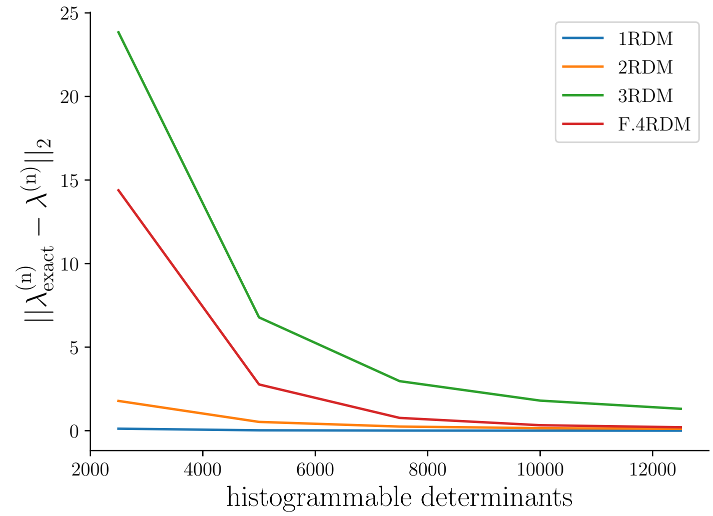

Figure S1: Convergence in the L_2 norm of the eigenvalues of CASPT2 intermediates obtained with histogramming to results from exact diagonalisation. Data was generated with the natural orbitals of the Cr_2 valence CASSCF(12,12) at 2.1 Å distance using 1×10^6 N_{walker} and variable semistochastic/histogrammable space sizes. The Fock matrix for the Fock-contracted 4RDM (F.4RDM) was taken from exact diagonalisation. That the F.4RDM converges at similar rates to the 3RDM, but with a smaller absolute errors, hints at advantageous error cancellations in the weighted sum over off-diagonals of the 4RDM, $\sum_{vx} f_{vx} \Gamma^{(4)}_{pq,rs,tu,vx}$.

S3 Input files

All orbital files are provided in plain text. The `cu2o2_bismuoxo_*` input files contain further comments explaining the general calculation setup.

S3.1 Hydrogenase Model $[NiFe]^{2-}$

S3.1.1 Singlet

Geometry

```
16

Ni 0.00000000 1.54056551 0.03183031
S -1.52720904 3.01011671 -0.63017631
S 1.52720904 3.01011671 -0.63017631
S 1.44298795 0.08199607 0.84797267
S -1.44298795 0.08199607 0.84797267
Fe 0.00000000 -1.33450141 -0.18441717
C 1.37197161 -2.01566924 -1.29432067
C -1.37197161 -2.01566924 -1.29432067
N 2.25575655 -2.41656086 -1.95108292
N -2.25575655 -2.41656086 -1.95108292
C 0.00000000 -2.71578994 0.77684404
O 0.00000000 -3.66744155 1.45367937
H -2.68882687 2.37511900 -0.32466517
H 2.68882687 2.37511900 -0.32466517
H -2.50467296 0.08638050 -0.00754388
H 2.50467296 0.08638050 -0.00754388
```

OpenMolcas

```
&gateway
    coord = $CurrDir/nife_s0.xyz
    basis
    Ni.ANO-RCC...7S6P5D3F2G1H
    basis
    Fe.ANO-RCC...7S6P5D3F2G1H
    basis
    H.ANO-RCC...3S1P
    basis
    ano-rcc-vtzp
    group = full
    ricd
    cdthreshold = 1.0d-6

&seward

&rasscf
    cionly
    fileorb = $CurrDir/nife_s0.LocOrb
    ciroot = 1 1 1
    spin = 1
    nactel = 20 0 0
```

```
    inactive = 44 28
    ras2 =      12  9
    symmetry = 1
    thrs = 1.0e-5 1.0e-1 5.0e-4
    mcm7
    totalwalkers = 20000
    rdmlinspace  = 20000 10 100
    ndpt
>>> copy fockdump.h5 $CurrDir/fockdump.h5

&caspt2
    fciqmc
    ndia
    nord
    * 23/24 are Ni1 and Fe1 3s
    frozen = 22 14
    multistate = 1 1
    ipea = 0.25
    imag = 0.1
```

Histogrammable set

```
reference:
  mbf_init:
    fermion: [
        # 11 11 11 11 11 11 00 00 00 00 00 00 11 11 11 11 00 00 00 00 00
        [0, 1, 2, 3, 4, 5, 12, 13, 14, 15,   21, 22, 23, 24, 25, 26, 33,
   34, 35, 36],
    ]
particles:
  ms2: 0
shift:
  damp: 0.5
  nw_targets: [50e6]
stats:
  period: 5
wavefunction:
  nw_init: 50000000
  load: yes
  save: yes
  large_ci_set:
    path: M7.large.h5
    ncycle_thresh: 5
    av_weight_thresh: 1
    delay: 100
propagator:
  tau_init: 0.002
  static_tau: yes
  stochastic: yes
  static_probs: no
  nadd: 3.0
  ncycle: 2000
  semistochastic:
    size: 20000
```

```
    delay: 0
hamiltonian:
  fermion:
    fcidump:
      path: nife_s0.FciDmp.h5
```

Histogramming

```
reference:
  mbf_init:
    fermion: [
        # 11 11 11 11 11 11 00 00 00 00 00 00 11 11 11 11 00 00 00 00 00
        [0, 1, 2, 3, 4, 5, 12, 13, 14, 15,   21, 22, 23, 24, 25, 26, 33, 34, 35, 36],
    ]
particles:
  ms2: 0
shift:
  damp: 0.5
  nw_targets: [50e6]
stats:
  period: 5
wavefunction:
  nw_init: 50000000
  load: yes
  load_large_ci: yes
  save_hist: yes
propagator:
  tau_init: 0.002
  static_tau: yes
  stochastic: yes
  static_probs: no
  nadd: 3.0
  ncycle: 10000
  semistochastic:
    size: 20000
    delay: 0
hamiltonian:
  fermion:
    fcidump:
      path: nife_s0.FciDmp.h5
```

CASPT2 intermediates

```
reference:
  mbf_init:
    fermion: [
        # 11 11 11 11 11 11 00 00 00 00 00 00 11 11 11 11 00 00 00 00 00
        [0, 1, 2, 3, 4, 5, 12, 13, 14, 15,   21, 22, 23, 24, 25, 26, 33, 34, 35, 36],
    ]
particles:
  ms2: 0
shift:
  damp: 0.5
```

```
  nw_targets: [50e6]
stats:
  period: 5
wavefunction:
  nw_init: 50000000
  load_large_ci:
    path: M7.large.h5
  load:
    path: M7.hist.h5
propagator:
  tau_init: 0.002
  static_tau: yes
  stochastic: yes
  static_probs: no
  nadd: 3.0
  ncycle: 0
  semistochastic:
    size: 20000
    delay: 0
hamiltonian:
  fermion:
    fcidump:
      path: nife_s0.FciDmp.h5
mae:
  notf_fill_discard_thresh: 0.5
  filling_algorithm: caspt2
  delay: 0
  rdm:
    save: yes
    ranks: [1, 3]
    spinfree: yes
    fock_4rdm:
      fock_path: fockdump.h5
  ncycle: 0
```

S3.1.2 Triplet

Geometry

```
16

Ni 0.08569137 0.00000000 -0.31415580
S 1.95701731 0.00000000 0.99659688
S 1.11444389 0.00000000 -2.30551088
S -1.39724178 -1.49473685 0.66102212
S -1.39724178 1.49473685 0.66102212
Fe -2.98896573 0.00000000 -0.04598786
C -3.90860983 -1.36203992 -0.97297485
C -3.90860983 1.36203992 -0.97297485
N -4.43888086 -2.23363951 -1.54927881
N -4.43888086 2.23363951 -1.54927881
C -4.10796917 0.00000000 1.20244040
O -4.88662994 0.00000000 2.07111541
```

```
H 1.36254796 0.00000000 2.21647492
H -0.01731372 0.00000000 -3.05649150
H -1.56717852 -2.52026569 -0.21700923
H -1.56717852 2.52026569 -0.21700923
```

OpenMolcas

```
&gateway
    coord = $CurrDir/nife_s1.xyz
    basis
    Ni.ANO-RCC...7S6P5D3F2G1H
    basis
    Fe.ANO-RCC...7S6P5D3F2G1H
    basis
    H.ANO-RCC...3S1P
    basis
    ano-rcc-vtzp
    group = full
    ricd
    cdthreshold = 1.0d-6

&seward

&rasscf
    cionly
    fileorb = $CurrDir/nife_s1.LocOrb
    ciroot = 1 1 1
    spin = 3
    nactel = 22 0 0
    inactive = 48 23
    ras2 =      13  9
    symmetry = 2
    thrs = 1.0e-5 1.0e-1 5.0e-4
    mcm7
    ndpt
    totalwalkers = 20000
    rdmlinspace  = 20000 10 100
>>> copy fockdump.h5 $CurrDir/fockdump.h5

&caspt2
    fciqmc
    ndia
    nord
    * 26/27 are Ni1 and Fe1 3s
    frozen = 25 11
    multistate = 1 1
    ipea = 0.25
    imag = 0.1
```

Histogrammable set

```
reference:
  mbf_init:
    fermion: [
```

```
        # 11 11 11 11 11 11 10 00 00 00 00 00 00 11 11 11 11 10 00 00 00 00
        [0, 1, 2, 3, 4, 5, 6, 13, 14, 15, 16, 17,   22, 23, 24, 25, 26, 27, 35, 36, 37, 38],
    ]
particles:
  ms2: 2
shift:
  damp: 0.5
  nw_targets: [50e6]
stats:
  period: 5
wavefunction:
  nw_init: 50000000
  load: yes
  save: yes
  large_ci_set:
    path: M7.large.h5
    ncycle_thresh: 5
    av_weight_thresh: 1
    delay: 100
propagator:
  tau_init: 0.002
  static_tau: yes
  stochastic: yes
  static_probs: no
  nadd: 3.0
  ncycle: 2000
  semistochastic:
    size: 20000
    delay: 0
hamiltonian:
  fermion:
    fcidump:
      path: nife_s1.FciDmp.h5
```

Histogramming

```
reference:
  mbf_init:
    fermion: [
        # 11 11 11 11 11 11 10 00 00 00 00 00 00 11 11 11 11 10 00 00 00 00
        [0, 1, 2, 3, 4, 5, 6, 13, 14, 15, 16, 17,   22, 23, 24, 25, 26, 27, 35, 36, 37, 38],
    ]
particles:
  ms2: 2
shift:
  damp: 0.5
  nw_targets: [50e6]
stats:
  period: 5
wavefunction:
```

```
  nw_init: 50000000
  load: yes
  load_large_ci: yes
  save_hist: yes
propagator:
  tau_init: 0.002
  static_tau: yes
  stochastic: yes
  static_probs: no
  nadd: 3.0
  ncycle: 10000
  semistochastic:
    size: 20000
    delay: 0
hamiltonian:
  fermion:
    fcidump:
      path: nife_s1.FciDmp.h5
```

CASPT2 intermediates

```
reference:
  mbf_init:
    fermion: [
        # 11 11 11 11 11 11 10 00 00 00 00 00 00 11 11 11 11 10 00 00 00 00
        [0, 1, 2, 3, 4, 5, 6, 13, 14, 15, 16, 17,   22, 23, 24, 25, 26, 27, 35, 36, 37, 38],
    ]
particles:
  ms2: 2
shift:
  damp: 0.5
  nw_targets: [50e6]
stats:
  period: 5
wavefunction:
  nw_init: 50000000
  load_large_ci:
    path: M7.large.h5
  load:
    path: M7.hist.h5
propagator:
  tau_init: 0.002
  static_tau: yes
  stochastic: yes
  static_probs: no
  nadd: 3.0
  ncycle: 0
  semistochastic:
    size: 20000
    delay: 0
hamiltonian:
  fermion:
```

```
    fcidump:
      path: nife_s1.FciDmp.h5
mae:
  notf_fill_discard_thresh: 0.5
  filling_algorithm: caspt2
  delay: 0
  rdm:
    save: yes
    ranks: [1, 2, 3]
    spinfree: yes
    fock_4rdm:
      fock_path: fockdump.h5
  ncycle: 0
```

S3.2 Copper Oxide $[(CuNH_3)]_2\,{O_2}^{2+}$

S3.2.1 Bis(μ-oxo)

Geometry

```
12
From: https://pubs.acs.org/doi/suppl/10.1021/acs.jctc.6b00714/suppl_file/
   ct6b00714_si_001.pdf
Cu 0.000000 1.400000 0.000000
Cu 0.000000 -1.400000 0.000000
O 0.000000 0.000000 1.150000
O 0.000000 0.000000 -1.150000
N 0.000000 3.400000 0.000000
N 0.000000 -3.400000 0.000000
H -0.939693 3.742020 0.000000
H 0.939693 -3.742020 0.000000
H 0.469846 3.742020 0.813798
H -0.469846 -3.742020 -0.813798
H 0.469846 3.742020 -0.813798
H -0.469846 -3.742020 0.813798
```

OpenMolcas

```
&gateway
    coord = $CurrDir/cu2o2_bismuoxo.xyz
    basis = CU.ANO-RCC...7S6P5D3F2G1H, H.ANO-RCC...3S1P, ano-rcc-vtzp
    group = full
    ricd
    cdthreshold = 1.0d-6

&seward

&rasscf
    fileorb = $CurrDir/cu2o2_bismuoxo.LocOrb
    cionly
    ciroot = 1 1 1
    spin = 1
    nactel = 24 0 0
```

```
    ras2 =      7  5  5  7
    inactive = 13  4  5 12
    * activate M7 interface
    mcm7
    * dump fock matrix in same orbital basis as CASCI
    ndpt
    totalwalkers = 20000
    rdmlinspace  = 20000 10 100
>>> copy fockdump.h5 $CurrDir/fockdump.h5

&caspt2
    * activate FCIQMC interface
    fciqmc
    * transform PT2 intermediates from arbitrary into pseudo-canonical
   orbitals
    ndia
    * convert pre-contracted F4RDM to normal order
    nord
    frozen = 6   1   2   5
    multistate = 1 1
    ipea = 0.25
    imag = 0.1
```

Histogrammable set

```
reference:
  mbf_init:
    fermion: [[0, 1, 2, 3, 7, 8, 12, 13, 17, 18, 19, 20, 24, 25, 26, 27,
   31, 32, 36, 37, 41, 42, 43, 44]]
particles:
  ms2: 0
shift:
  init: 0.2
  nw_targets: [100e6]
stats:
  period: 5
wavefunction:
  nw_init: 100000000
  load: yes
  # select the set to be histogrammed in later calculations
  large_ci_set:
    path: M7.large.h5
    ncycle_thresh: 5
    av_weight_thresh: 1
    delay: 100
    max_size: 100000000
propagator:
  tau_init: 0.001
  static_tau: yes
  stochastic: yes
  static_probs: no
  nadd: 3.0
  ncycle: 2000
  semistochastic:
```

```
    size: 20000
    delay: 0
hamiltonian:
  fermion:
    fcidump:
      path: bismuoxo.FciDmp.h5
```

Histogramming

```
reference:
  mbf_init:
    fermion: [[0, 1, 2, 3, 7, 8, 12, 13, 17, 18, 19, 20, 24, 25, 26, 27,
   31, 32, 36, 37, 41, 42, 43, 44]]
particles:
  ms2: 0
shift:
  init: 0.3
  nw_targets: [100e6]
stats:
  period: 5
wavefunction:
  nw_init: 100000000
  # load an arbitrary wave function to start from
  load:
    path: M7.wf.h5
  # the determinants in this file will be histogrammed ...
  load_large_ci:
    path: M7.large.h5
  # ... and their averaged coefficients written to this file
  save_hist:
    path: M7.hist.h5
propagator:
  tau_init: 0.001
  static_tau: yes
  stochastic: yes
  static_probs: no
  nadd: 3.0
  ncycle: 11000
  semistochastic:
    size: 20000
    delay: 0
hamiltonian:
  fermion:
    fcidump:
      path: bismuoxo.FciDmp.h5
```

CASPT2 intermediates

```
reference:
  mbf_init:
    fermion: [[0, 1, 2, 3, 7, 8, 12, 13, 17, 18, 19, 20, 24, 25, 26, 27,
   31, 32, 36, 37, 41, 42, 43, 44]]
particles:
  ms2: 0
shift:
```

```
  damp: 0.5
  nw_targets: [100e6]
stats:
  period: 5
wavefunction:
  # the histogrammed wave function will be rescaled to this value
  # affects the meaning of notf_fill_discard_thresh
  nw_init: 100000000
  # determinants from M7.hist.h5 to be used for RDM computation
  load_large_ci:
    path: M7.large.h5
  # read the average wave function to start from
  load:
    path: M7.hist.h5
propagator:
  tau_init: 0.001
  static_tau: yes
  stochastic: yes
  static_probs: no
  nadd: 3.0
  # perform no propagation
  ncycle: 0
  semistochastic:
    size: 10000
    delay: 0
hamiltonian:
  fermion:
    fcidump:
      path: bismuoxo.FciDmp.h5
mae:
  # discard determinants will avg weight less than 1.0
  notf_fill_discard_thresh: 1.0
  # use a histogrammed wave function for CASPT2 intermediates
  filling_algorithm: caspt2
  # do not delay RDM formation
  delay: 0
  rdm:
    save: yes
    ranks: [1, 2, 3]
    spinfree: yes
    # only required for F4RDM
    fock_4rdm:
      fock_path: fockdump.h5
  # do not perform sampling
  ncycle: 0
```

S3.2.2 Peroxo

Geometry

```
12
From: https://pubs.acs.org/doi/suppl/10.1021/acs.jctc.6b00714/suppl_file/
   ct6b00714_si_001.pdf
```

```
Cu 0.000000 1.800000 0.000000
Cu 0.000000 -1.800000 0.000000
O 0.000000 0.000000 0.700000
O 0.000000 0.000000 -0.700000
N 0.000000 3.800000 0.000000
N 0.000000 -3.800000 0.000000
H -0.939693 4.142020 0.000000
H 0.939693 -4.142020 0.000000
H 0.469846 4.142020 0.813798
H -0.469846 -4.142020 -0.813798
H 0.469846 4.142020 -0.813798
H -0.469846 -4.142020 0.813798
```

OpenMolcas

```
&gateway
    coord = $CurrDir/cu2o2_peroxo.xyz
    basis = CU.ANO-RCC...7S6P5D3F2G1H, H.ANO-RCC...3S1P, ano-rcc-vtzp
    group = full
    ricd
    cdthreshold = 1.0d-6

&seward

&rasscf
    fileorb = $CurrDir/cu2o2_peroxo.LocOrb
    cionly
    ciroot = 1 1 1
    spin = 1
    nactel = 24 0 0
    ras2 =      7  5  5  7
    inactive = 13  4  5 12
    mcm7
    ndpt
    totalwalkers = 20000
    rdmlinspace  = 20000 10 100
>>> copy fockdump.h5 $CurrDir/fockdump.h5

&caspt2
    fciqmc
    ndia
    nord
    frozen = 6  1  2  5
    multistate = 1 1
    ipea = 0.25
    imag = 0.1
```

Histogrammable set

```
reference:
  mbf_init:
    fermion: [[0, 1, 2, 3, 7, 8, 12, 13, 17, 18, 19, 20, 24, 25, 26, 27,
    31, 32, 36, 37, 41, 42, 43, 44]]
particles:
  ms2: 0
```

```
shift:
  init: 0.2
  nw_targets: [100e6]
stats:
  period: 5
wavefunction:
  nw_init: 100000000
  load: yes
  large_ci_set:
    path: M7.large.h5
    ncycle_thresh: 5
    av_weight_thresh: 1
    delay: 100
    max_size: 100000000
propagator:
  tau_init: 0.001
  static_tau: yes
  stochastic: yes
  static_probs: no
  nadd: 3.0
  ncycle: 2000
  semistochastic:
    size: 20000
    delay: 0
hamiltonian:
  fermion:
    fcidump:
      path: peroxo.FciDmp.h5
```

Histogramming

```
reference:
  mbf_init:
    fermion: [[0, 1, 2, 3, 7, 8, 12, 13, 17, 18, 19, 20, 24, 25, 26, 27,
    31, 32, 36, 37, 41, 42, 43, 44]]
particles:
  ms2: 0
shift:
  init: 0.2
  nw_targets: [100e6]
stats:
  period: 5
wavefunction:
  nw_init: 100000000
  load:
    path: M7.wf.h5
  load_large_ci:
    path: M7.large.h5
  save_hist:
    path: M7.hist.h5
propagator:
  tau_init: 0.001
  static_tau: yes
  stochastic: yes
```

```
  static_probs: no
  nadd: 3.0
  ncycle: 11000
  semistochastic:
    size: 20000
    delay: 0
hamiltonian:
  fermion:
    fcidump:
      path: peroxo.FciDmp.h5
```

CASPT2 intermediates

```
reference:
  mbf_init:
    fermion: [[0, 1, 2, 3, 7, 8, 12, 13, 17, 18, 19, 20, 24, 25, 26, 27, 31, 32, 36, 37, 41, 42, 43, 44]]
particles:
  ms2: 0
shift:
  damp: 0.5
  nw_targets: [100e6]
stats:
  period: 5
wavefunction:
  nw_init: 100000000
  load_large_ci:
    path: M7.large.h5
  load:
    path: M7.hist.h5
propagator:
  tau_init: 0.001
  static_tau: yes
  stochastic: yes
  static_probs: no
  nadd: 3.0
  ncycle: 0
  semistochastic:
    size: 10000
    delay: 0
hamiltonian:
  fermion:
    fcidump:
      path: peroxo.FciDmp.h5
mae:
  notf_fill_discard_thresh: 1.0
  filling_algorithm: caspt2
  delay: 0
  rdm:
    save: yes
    ranks: [1, 3]
    spinfree: yes
    fock_4rdm:
      fock_path: fockdump.h5
```

```
  ncycle: 0
```

S3.3 Fe-Porphyrin Model Fe(P)

Geometry

```
   29

Fe      0.000000      0.000000      0.000000
 N      1.406727      1.406727      0.000000
 N     -1.406727      1.406727      0.000000
 N      1.406727     -1.406727      0.000000
 N     -1.406727     -1.406727      0.000000
 C     -0.000000      3.400142      0.000000
 C     -0.000000     -3.400142      0.000000
 C      3.400142     -0.000000      0.000000
 C     -3.400142     -0.000000      0.000000
 C      1.222770      2.760387      0.000000
 C     -1.222770      2.760387      0.000000
 C      1.222770     -2.760387      0.000000
 C     -1.222770     -2.760387      0.000000
 C      2.760387      1.222770      0.000000
 C     -2.760387      1.222770      0.000000
 C      2.760387     -1.222770      0.000000
 C     -2.760387     -1.222770      0.000000
 H      0.000000      4.482672      0.000000
 H      0.000000     -4.482672      0.000000
 H      4.482672      0.000000      0.000000
 H     -4.482672      0.000000      0.000000
 H      2.181081      3.277651      0.000000
 H     -2.181081      3.277651      0.000000
 H      2.181081     -3.277651      0.000000
 H     -2.181081     -3.277651      0.000000
 H      3.277651      2.181081      0.000000
 H     -3.277651      2.181081      0.000000
 H      3.277651     -2.181081      0.000000
 H     -3.277651     -2.181081      0.000000
```

S3.3.1 Triplet

OpenMolcas

```
&gateway
  ricd
  coord = $CurrDir/fepor_s1.xyz
  basis = ano-rcc-vtzp
  group = full

&seward

&rasscf
    cionly
```

```
    fileorb = $CurrDir/fepor_s1.LocOrb
    spin = 3
    symmetry = 6
    nactel = 26 0 0
    inactive = 17 14 14 9 2 0 0 0
    ras2 =      4  0  0 3 5 6 6 3
    thrs = 1.0e-4 1.0e-1 5.0e-4
    mcm7
    ndpt
    totalwalkers = 20000
    rdmlinspace  = 20000 10 100
* >>> copy fockdump.h5 $CurrDir/fockdump.h5

&caspt2
    fciqmc
    ndia
    nord
    froz = 7 5 5 3 1 0 0 0
    multistate = 1 1
    ipea = 0.25
    imag = 0.1
```

Histogrammable set

```
reference:
  mbf_init:
            # 11 10 00 00   11 00 00   11 11 11 00 00   11 11 10 00 00 00
      11 11 11 00 00 00   11 11 00
    fermion: [[ 0, 1, 4, 7, 8, 9, 12, 13, 14, 18, 19, 20, 24, 25, 27, 31,
   34, 35, 36, 39, 40, 45, 46, 47, 51, 52]]
particles:
  ms2: 2
shift:
  damp: 0.3
  nw_targets: [125e6]
stats:
  period: 5
wavefunction:
  nw_init: 125000000
  load: yes
  save: yes
  large_ci_set:
    path: M7.large.h5
    ncycle_thresh: 5
    av_weight_thresh: 1
    delay: 100
    max_size: 150000000
propagator:
  tau_init: 0.002
  static_tau: yes
  stochastic: yes
  static_probs: no
  nadd: 3.0
  ncycle: 1500
```

```
  semistochastic:
    size: 20000
    delay: 0
hamiltonian:
  fermion:
    fcidump:
      path: tripl.FciDmp.h5
```

Histogramming

```
reference:
  mbf_init:
            # 11 10 00 00   11 00 00   11 11 11 00 00   11 11 10 00 00 00
     11 11 11 00 00 00   11 11 00
    fermion: [[ 0, 1, 4, 7, 8, 9, 12, 13, 14, 18, 19, 20, 24, 25, 27, 31,
   34, 35, 36, 39, 40, 45, 46, 47, 51, 52]]
particles:
  ms2: 2
shift:
  damp: 0.3
  nw_targets: [125e6]
stats:
  period: 5
wavefunction:
  nw_init: 125000000
  load: yes
  load_large_ci: yes
  save_hist: yes
propagator:
  tau_init: 0.002
  static_tau: yes
  stochastic: yes
  static_probs: no
  nadd: 3.0
  ncycle: 30000
  semistochastic:
    size: 20000
    delay: 0
hamiltonian:
  fermion:
    fcidump:
      path: tripl.FciDmp.h5
```

CASPT2 intermediates

```
reference:
  mbf_init:
            # 11 10 00 00   11 00 00   11 11 11 00 00   11 11 10 00 00 00
     11 11 11 00 00 00   11 11 00
    fermion: [[ 0, 1, 4, 7, 8, 9, 12, 13, 14, 18, 19, 20, 24, 25, 27, 31,
   34, 35, 36, 39, 40, 45, 46, 47, 51, 52]]
particles:
  ms2: 2
shift:
  damp: 0.3
```

```
  nw_targets: [125e6]
stats:
  period: 5
wavefunction:
  nw_init: 125000000
  load:
    path: M7.hist.h5
  load_large_ci: yes
propagator:
  tau_init: 0.002
  static_tau: yes
  stochastic: yes
  static_probs: no
  nadd: 3.0
  ncycle: 0
  semistochastic:
    size: 20000
    delay: 0
hamiltonian:
  fermion:
    fcidump:
      path: tripl.FciDmp.h5
mae:
  notf_fill_discard_thresh: 2.0
  filling_algorithm: caspt2
  delay: 0
  rdm:
    save: yes
    ranks: [1, 3]
    spinfree: yes
    fock_4rdm:
      fock_path: fockdump.h5
  ncycle: 0
```

S3.3.2 Quintet

OpenMolcas

```
&gateway
  ricd
  coord = $CurrDir/fepor_s2.xyz
  basis = ano-rcc-vtzp
  group = full

&seward

&rasscf
    cionly
    fileorb = $CurrDir/fepor_s2.LocOrb
    spin = 5
    symmetry = 1
    nactel = 26 0 0
    inactive = 17 14 14 9 2 0 0 0
```

```
    ras2 =      4  0  0 3 5 6 6 3
    thrs = 1.0e-4 1.0e-1 5.0e-4
    mcm7
    ndpt
    totalwalkers = 20000
    rdmlinspace  = 20000 10 100
>>> copy fockdump.h5 $CurrDir/fockdump.h5

&caspt2
    fciqmc
    ndia
    nord
    froz = 7 5 5 3 1 0 0 0
    multistate = 1 1
    ipea = 0.25
    imag = 0.1
```

Histogrammable set

```
reference:
  mbf_init:
    fermion: [
        [0, 1, 4, 5, 7, 8, 9, 12, 13, 14, 18, 19, 20, 24, 25, 27, 31, 34,
   35, 36, 39, 40, 45, 46, 51, 52],
    ]
particles:
  ms2: 4
shift:
  damp: 0.3
  nw_targets: [125e6]
stats:
  period: 5
wavefunction:
  nw_init: 125000000
  load: yes
  save: yes
  large_ci_set:
    path: M7.large.h5
    ncycle_thresh: 5
    av_weight_thresh: 1
    delay: 100
    max_size: 150000000
propagator:
  tau_init: 0.002
  static_tau: yes
  stochastic: yes
  static_probs: no
  nadd: 3.0
  ncycle: 1500
  semistochastic:
    size: 20000
    delay: 0
hamiltonian:
  fermion:
```

```
    fcidump:
      path: quint.FciDmp.h5
```

Histogramming

```
reference:
  mbf_init:
    fermion: [
        [0, 1, 4, 5, 7, 8, 9, 12, 13, 14, 18, 19, 20, 24, 25, 27, 31, 34,
   35, 36, 39, 40, 45, 46, 51, 52],
    ]
particles:
  ms2: 4
shift:
  damp: 0.3
  nw_targets: [125e6]
stats:
  period: 5
wavefunction:
  nw_init: 125000000
  load: yes
  load_large_ci: yes
  save_hist: yes
propagator:
  tau_init: 0.002
  static_tau: yes
  stochastic: yes
  static_probs: no
  nadd: 3.0
  ncycle: 30000
  semistochastic:
    size: 20000
    delay: 0
hamiltonian:
  fermion:
    fcidump:
      path: quint.FciDmp.h5
```

CASPT2 intermediates

```
reference:
  mbf_init:
    fermion: [
        [0, 1, 4, 5, 7, 8, 9, 12, 13, 14, 18, 19, 20, 24, 25, 27, 31, 34,
   35, 36, 39, 40, 45, 46, 51, 52],
    ]
particles:
  ms2: 4
shift:
  damp: 0.3
  nw_targets: [125e6]
stats:
  period: 5
wavefunction:
  nw_init: 125000000
```

```
  load:
    path: M7.hist.h5
  load_large_ci: yes
propagator:
  tau_init: 0.002
  static_tau: yes
  stochastic: yes
  static_probs: no
  nadd: 3.0
  ncycle: 0
  semistochastic:
    size: 20000
    delay: 0
hamiltonian:
  fermion:
    fcidump:
      path: quint.FciDmp.h5
mae:
  notf_fill_discard_thresh: 1.0
  filling_algorithm: caspt2
  delay: 0
  rdm:
    save: yes
    ranks: [1]
    spinfree: yes
    fock_4rdm:
      fock_path: fockdump.h5
  ncycle: 0
```
